# Utilization of point-of-care ultrasound and rotational thromboelastometry (ROTEM) in the diagnosis and management of amniotic fluid embolism presenting as post-partum hemorrhage and cardiac arrest

**DOI:** 10.1515/crpm-2022-0009

**Published:** 2022-08-15

**Authors:** Angela N. Phillips, Lisa L. Kirkland, William E. Wagner, Roman Melamed, David M. Tierney

**Affiliations:** Department of Graduate Medical Education, Abbott Northwestern Hospital, Allina Health, Minneapolis, MN, USA; Department of Critical Care, Abbott Northwestern Hospital, Allina Health, Minneapolis, MN, USA; Departments of Maternal Fetal Medicine and Critical Care, Abbott Northwestern Hospital, Allina Health, Minneapolis, MN, USA

**Keywords:** amniotic fluid embolism, cardiac arrest, disseminated intravascular coagulation, labor and delivery, point-of-care ultrasonography, pregnancy

## Abstract

**Objectives:**

To describe the integration of point-of-care ultrasound (POCUS) and rotational thromboelastometry (ROTEM) in the diagnosis and management of cardiac arrest secondary to amniotic fluid embolism (AFE).

**Case presentation:**

A 29-year-old female presented for induction of labor at 39 weeks. Labor was complicated by hemorrhage and subsequent sinus tachycardia pulseless electrical activity (PEA) arrest. Intra-arrest POCUS demonstrated right ventricular dilation and hypokinesis adding to a presumed hemorrhagic arrest etiology. Venoarterial extracorporeal membrane oxygenation (VA-ECMO) was initiated at the bedside following the POCUS findings. ROTEM further clarified the etiology of hemorrhage as disseminated intravascular coagulation (DIC), and in combination with the POCUS findings led to a final diagnosis of amniotic fluid embolism with DIC. The patient was maintained on VA-ECMO without heparin in the setting of DIC. She had a complicated hospital course but was discharged home with her healthy child and no residual physical or neurologic deficits.

**Conclusions:**

In the absence of more specific testing modalities the utilization of rapidly available POCUS in conjunction with ROTEM can impact clinical decision making of cardiovascular resuscitation in patients during labor and delivery by narrowing the differential between pulmonary embolism and AFE.

## Introduction

Amniotic fluid embolism (AFE) is a difficult to predict, rare and life-threatening obstetrical emergency. The nonspecific clinical manifestations, poorly defined diagnostic criteria and rapid clinical progression all contribute to its high mortality rate documented at 11–61% [1], [2], [3], [4], [5]. Analysis of the US registry for AFE estimates a mortality of 56% in the first 2 h (61% overall) emphasizing the importance of rapid diagnosis [3, 5]. However, diagnosis of AFE remains difficult and is often a clinical diagnosis of exclusion given the lack of specific diagnostic modalities [2, 3].

Point-of-care ultrasound (POCUS) has a well-defined role in the fields of emergency and critical care medicine [6, 7]. However, it remains underutilized in the diagnosis and management of obstetrical emergencies, specifically AFE. Though not specific, right ventricular (RV) dysfunction on POCUS can narrow the differential of circulatory collapse in the post-partum patient to a few entities including, but not limited to, AFE and pulmonary embolism (PE) [6]. However, further differentiation between AFE and PE remains vital as timely administration of tissue plasminogen activator (tPA) for PE can improve outcomes, whereas inappropriate administration in AFE can worsen bleeding among the 30–45% of patients with AFE who develop disseminated intravascular coagulation (DIC) [3]. Subsequently, the combined utilization of POCUS and ROTEM can further distinguish AFE from PE and direct treatment by identifying DIC [3, 8].

ROTEM is a point-of-care viscoelastic assay used to evaluate the properties of blood coagulation from clot formation to lysis [2, 9]. ROTEM has been shown to reduce transfusion related morbidity in cardiovascular surgery. However, the benefit of ROTEM in obstetrical complications remained uncertain until recently when its potential benefit in the management of post-partum hemorrhage was demonstrated [8, 9]. Additionally, there have been reported cases, including the current presented case, that have highlighted the benefit of using ROTEM in the diagnosis and management of AFE, specifically in identifying DIC and directing blood product transfusion [2, 8, 10]. The pace at which ROTEM can generate valuable information about a patient’s coagulation properties has an undeniable advantage to conventional laboratory markers, which can positively impact the morbidity and mortality associated with AFE [2, 11].

We present a case where the utilization of POCUS in conjunction with ROTEM resulted in the rapid diagnosis and management of AFE with DIC leading to successful bedside venoarterial extracorporeal membrane oxygenation (VA-ECMO) initiation without the use of heparin and rapid resuscitation following cardiac arrest in a pregnant patient.

## Case presentation

A 29-year-old, previously healthy, gravida one female presented at 39 weeks gestation for induction of labor due to gestational diabetes and concerns surrounding the rapidly increasing local COVID-19 rate. Induction with misoprostol was complicated by late decelerations on fetal monitoring and a period of tachysystole due to frequent, difficult to manage, contractions. The patient was laboring for approximately 10 h when she suddenly felt nauseated, lightheaded, and subsequently suffered a syncopal episode complicated by fetal bradycardia. The patient was noted to be tachycardic, poorly responsive and in respiratory distress. She was assisted to the supine position, placed on oxygen, and was transferred to the operating room (OR) 6 min later for emergency Cesarean section (C-section).

Eight minutes after uterine incision, the intubated patient developed circulatory collapse followed by a sinus tachycardia with pulseless electrical activity (PEA) arrest. Manual chest compressions were initiated, and the hospital code/resuscitation team was called to the OR. The patient’s incision was closed emergently, and chest compressions were transitioned to a mechanical chest compression device.

Shortly after incision closure, the patient began oozing from her vascular access sites and through her surgical dressings. The hospital’s massive transfusion protocol was initiated with a diagnosis of hemorrhagic shock. After 17 min of arrest and volume resuscitation, the patient had ventricular tachycardia and was defibrillated three times, eventually returning to sinus tachycardia without a pulse. Return of spontaneous circulation with a systolic blood pressure of 60–70 mmHg was achieved after 6 L of saline and 8 units of blood products via bilateral tibial interosseous lines and an internal jugular catheter supplemented by vasopressors. The patient continued to bleed from vascular access sites and her incision.

**Figure 1:**
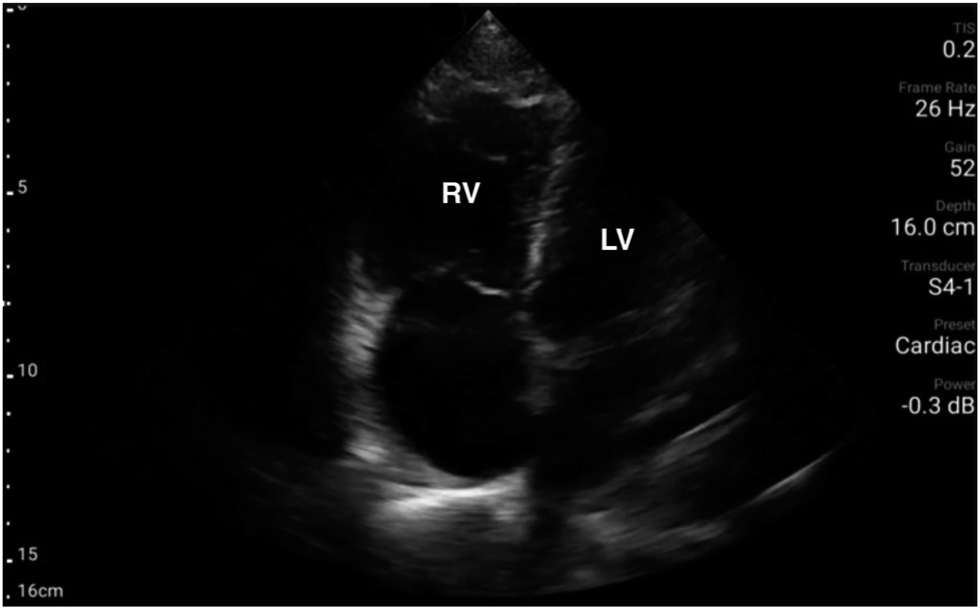
POCUS apical 4-chamber. Apical 4-chamber view of the heart at end-systole showing severely dilated and hypokinetic right ventricle (RV) and underfilled, hyperdynamic left ventricle (LV). See Video 1: Supplementary Digital Content 1 for video clip.

During this return of blood pressure, the code team, equipped with POCUS ability and equipment obtained an apical 4-chamber view (Figure 1, Video 1: Supplementary Digital Material 1) from the window available while the mechanical compression device remained in place. Ultrasound revealed a severely dilated and hypokinetic RV, and a hyperdynamic, underfilled left ventricle concerning for RV obstructive shock. This resulted in the possible diagnoses of PE or AFE being added to hemorrhagic shock. The combination of significant oozing and increased vaginal bleeding at this point brought AFE to the top of the differential as a unifying diagnosis, and the bedside extracorporeal membrane oxygenation (ECMO) team was activated. The patient successfully underwent VA-ECMO cannulation (right femoral vein/artery) at the bedside and was on flow 45 min following cardiac arrest (Video 2: Supplementary Digital Material 2).

**Figure 2:**
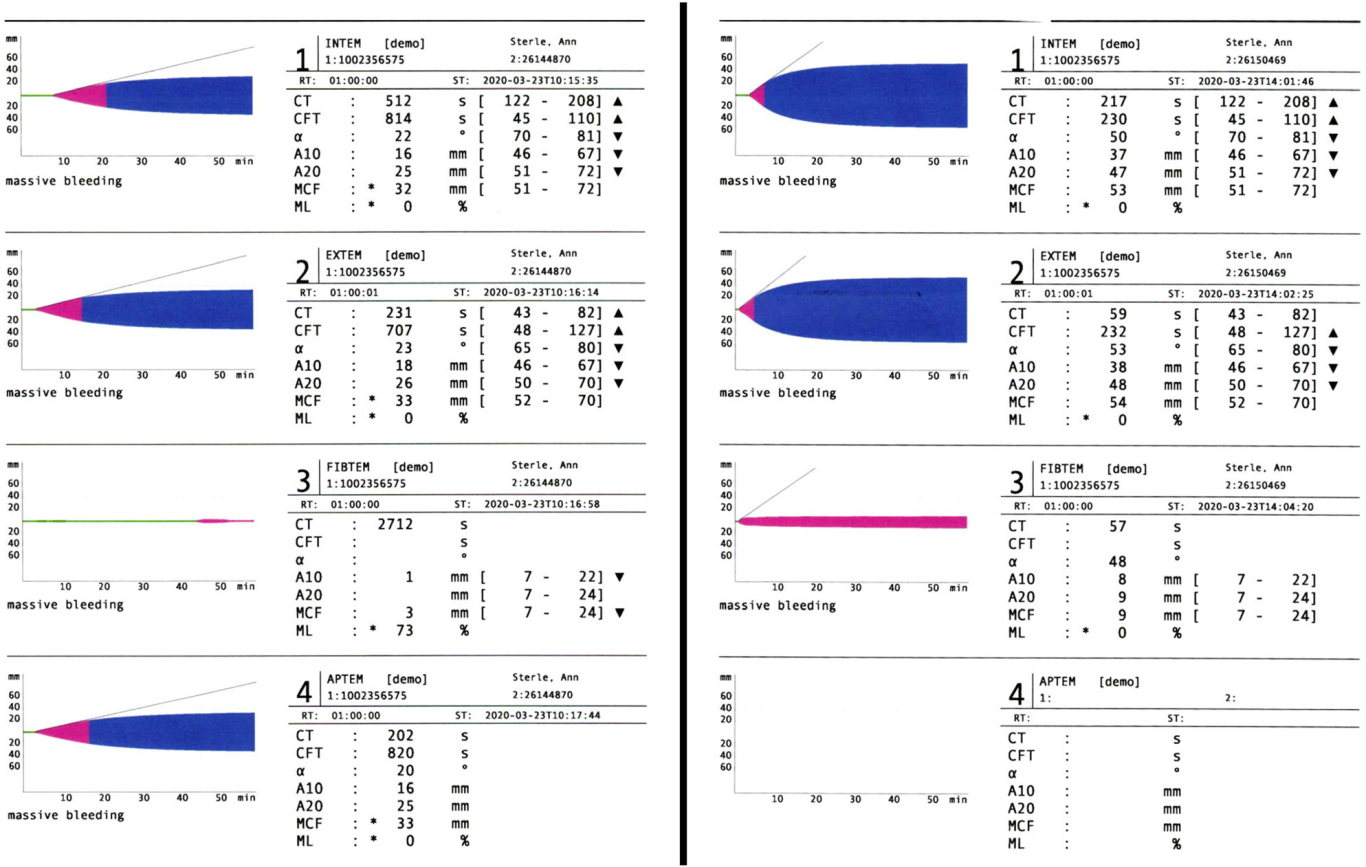
ROTEM results. Rotational thromboelastometry (ROTEM) results immediately following cardiac arrest (left) and follow-up analysis after initial stabilization and transfusion (right).

ROTEM demonstrated markedly prolonged clotting time, prolonged clotting formation time and very low clot amplitude suggestive of poor clot stability on INTEM and EXTEM. FIBTEM and APTEM showed low fibrin and fibrinogen clot contribution as well as premature lysis (Figure 2). ROTEM results consistent with DIC prompted continuation of the massive transfusion protocol and further reason to withhold heparin after VA-ECMO cannulation. The patient received eight units of red blood cells, two units of fresh frozen plasma, five units of cryoprecipitate and four units of platelets during initial stabilization for an estimated blood loss greater than 2.5 L.

Following VA-ECMO canulation and stabilization, patient was transferred to the cardiovascular intensive care unit with a diagnosis of AFE complicated by DIC and post-partum hemorrhage. Laboratory results following resuscitation showed a 5 g/dL drop in hemoglobin, a 90,000/mcL drop in platelets, low fibrinogen, and an elevated D-dimer, INR and aPTT further confirming the diagnosis of DIC (Table 1). Repeat ROTEM following resuscitation showed improvement in coagulopathy with normalization of clotting time and improved amplitude on INTEM and EXTEM with normalization of fibrin and fibrinogen levels on FIBTEM (Figure 2). Our lab did not report post-resuscitation APTEM values. She was removed from ECMO on ICU day 2.

**Table 1:** Laboratory values.

	Lab results prior to cardiac arrest	Lab results 30 min following cardiac arrest^a^	Lab results 90 min following cardiac arrest^b^
Hemoglobin, g/dL	13.0	9.0	11.2
Platelet count, thou/cu mm	–	171,000	81,000
D-Dimer, mcg/ml	–	–	>4.00
APTT, s	–	–	95
Fibrinogen, mg/dL	–	–	100
INR	–	–	2.5

Conventional laboratory values prior to cardiac arrest, during resuscitation, and following early stabilization. ^a^Labs values after 3 units of red blood cells, 1 units of platelets, 0 units of plasma and 2 units of cryoprecipitate; ^b^Lab values after an additional 5 units of red blood cells, 3 units of platelets, 2 units of plasma and 3 units of cryoprecipitate.

She had a reassuring neurologic exam on ICU day 0 where she followed commands. Brain CT on ICU day 1 showed a couple small regions of ischemia but no concern for anoxic brain injury with well-preserved grey/white matter. The patient’s early ICU course was complicated by prolonged acute encephalopathy with concern for seizure activity treated empirically with levetiracetam. Brain MRI on ICU day 3 showed small bilateral hemispheric strokes and a couple areas of tiny hemorrhage, but an intact cortical ribbon. Over the subsequent days her mentation significantly improved, and she was extubated on ICU day 18 with a tracheostomy in place and subsequently decannulated on day 22. She was discharged to acute care rehabilitation with mild residual right-sided weakness, difficulty with fine motor tasks and word finding. Six days later she was discharged home to her new healthy child with independent mobility and self-cares and a very mild expressive aphasia that had significantly improved.

## Discussion

Amniotic fluid embolism is a leading cause of maternal mortality world-wide. However, despite its clinical significance, there is little consensus among health care providers regarding the diagnosis. The American Society of Maternal-Fetal Medicine, The US registry of AFE, the United Kingdom obstetric surveillance system, and the Japanese AFE criteria have outlined several diagnostic criteria to aid diagnosis; however, given the lack of specific diagnostic modalities these criteria remain vague and provide little clinical guidance [12]. This case meets the diagnostic criteria for research reporting of AFE (sudden onset cardiorespiratory arrest, overt DIC, onset during labor or within 30 min of placental delivery, lack of fever during labor) set forth by Clark et al. [13]. The presented case also demonstrates that, in the absence of more specific diagnostic guidelines, POCUS in combination with ROTEM can be instrumental in differentiating between AFE and PE and guide appropriate management of AFE within this largely young and healthy patient population.

In this case, POCUS enabled the rapid identification of RV strain and obstructive shock which increased the suspicion for AFE and altered the resuscitation effort to address both hemorrhagic and obstructive shock, leading to VA-ECMO initiation. The use of POCUS during cardiovascular resuscitation in the setting of AFE is infrequently described. There are two other documented case reports describing the use of echocardiography to identify masses in the right ventricle with severely reduced right ventricular function leading to the diagnosis of AFE [14, 15]. Simard et al. outlined three cases where POCUS was used to identify the etiology of shock following circulatory collapse in the post-partum patient and further concluded that POCUS may be an important diagnostic criterion for AFE [16]. Though cardiac findings on ultrasound are not specific for AFE, they can guide clinical decision making by narrowing the differential diagnosis for circulatory collapse in the post- or peripartum patient.

ROTEM was readily utilized in this case and allowed for the rapid identification of severe coagulopathy consistent with DIC. One study estimated the occurrence of DIC in the setting of pulmonary embolism at 1% compared to 30–77% in AFE [17]. Therefore, the use of ROTEM in the setting of already identified right heart strain can help further differentiate between PE and AFE and guide appropriate life-saving treatment. Additional case reports have highlighted the usefulness of ROTEM in identifying DIC and guiding blood product transfusions [2, 8, 10]. Amgalan et al. reviewed several studies that showed improved clinical outcomes when transfusions, in the setting of post-partum hemorrhage, were guided by ROTEM. Studies also demonstrate a significant decrease in the number of unnecessary transfusions, hysterectomies, and post-partum ICU admissions when ROTEM was used in the management of post-partum hemorrhage [9]. Some studies have described an early hyperfibrinolysis stage of AFE that quickly resolves [12, 18, 19]. Our initial post-arrest ROTEM analysis did not demonstrate this stage if it existed, likely because of the time lapse of hours between onset of initial symptoms, performance of the C-section, the prolonged initial resuscitation in the OR, and then the initial ROTEM sample being collected. Critiques of ROTEM and other viscoelastic modalities claim poor study design and bias leading to inconsistent results regarding its clinical benefit [9]. Despite inconsistent data, ROTEM can be a valuable tool in the diagnosis of AFE in the setting of right heart strain as seen in the presented case.

The difficulty in identification and diagnosis of AFE is in part due to the lack of clarity and consensus regarding pathophysiology and risk factors. Initially, AFE was seen largely as a mechanical obstruction [3, 6]. However, later research demonstrated that amniotic fluid contains higher levels of hypercoagulable and vasoactive substances compared to maternal circulation which can cause a robust inflammatory reaction leading to hypoxemia, coagulopathy, and circulatory collapse [3, 5]. Tamura et al. describe both an inflammatory response and a mast cell mediated-anaphylactoid reaction that is independent of antigen-antibody-mediated classic anaphylaxis [20]. Rath and Dedhia describe AFE as a biphasic disease process. Phase one is marked by pulmonary vasoconstriction leading to pulmonary hypertension and right heart strain. Phase two is discernible by the development of DIC, possible left heart failure and pulmonary edema [3, 4]. This case further highlights this biphasic process with the patient developing hypoxemia, respiratory distress, and circulatory collapse, followed by the development of profound DIC.

Several risk factors for AFE have been identified [1, 4]. Rath et al. provide a condensed list of main risk factors which includes maternal age >35, C-section, placenta previa, induction of labor and multiple pregnancies. A few case reports including the presented case share several risk factors–namely induction of labor, difficult labor, and C-section [2, 4, 8]. However, despite several known risk factors, identifying AFE remains difficult largely due to the nonspecific clinical manifestations. As seen in the presented case, AFE may initially present with a nonspecific prodrome of presyncope, agitation and confusion [2, 10]. It has been estimated that cardiac arrest occurs in 30–87% of cases, acute dyspnea in 50–80% of cases, fetal distress in 20–36% of cases, and DIC in 30–77% of cases–all of which occurred in our patient. These manifestations cast a broad differential diagnosis including pulmonary embolism, septic shock, acute coronary syndrome, aortic dissection, hemorrhagic shock, peripartal cardiomyopathy, complication of anesthesia, post-partum hemorrhage, placental abruption, and uterine rupture [3]. However, as seen in this case ROTEM in combination with POCUS can quickly narrow this differential, help differentiate AFE from PE in setting of right heart strain and guide clinical decision making.

## Take home message

Providers must have a high level of suspicion for AFE in the peripartum or post-partum patient who suffers circulatory collapse. POCUS and ROTEM are valuable tools in diagnosis and resuscitation following cardiac arrest due to AFE and can have a positive impact on clinical outcomes by differentiating AFE from PE in the setting of right heart strain.

## Supplementary Material

Supplementary Material
