# Supplementary material for: Utilization of point-of-care ultrasound and rotational thromboelastometry (ROTEM) in the diagnosis and management of amniotic fluid embolism presenting as post-partum hemorrhage and cardiac arrest
Source: Case Rep Perinat Med. 2022 Aug 15;11(1):20220009. doi: 10.1515/crpm-2022-0009 (PMC11800665; doi:10.1515/crpm-2022-0009)
Supplement: Supplementary file 1 — Supplementary Material [file j_crpm-2022-0009_suppl.docx]

**SUPPLEMENTAL DIGITAL CONTENT**

**Supplemental Digital Content 1** (.mp4 video file; 0:04min; 741 KB; Author - David Tierney):  Apical 4-chamber view of the heart demonstrating severely dilated and hypokinetic right ventricle (RV) and underfilled, hyperdynamic left ventricle (LV).

**Supplemental Digital Content** 2 (.mp4 video file; 0:04min; 552 KB; Author - David Tierney):  Subcostal 4/5-chamber view of the heart demonstrating hypokinetic right and left ventricles and improvement in right ventricular dilation following initial resuscitation efforts and VA-ECMO cannulation/initiation at the bedside.
